# Supplementary material for: Role of Adatoms for the Adsorption of F4TCNQ on Au(111)
Source: J Phys Chem C Nanomater Interfaces. 2022 Apr 21;126(17):7718–27. doi: 10.1021/acs.jpcc.2c00994 (PMC9082607; doi:10.1021/acs.jpcc.2c00994)
Supplement: Supplementary file 1 — jp2c00994_si_001.pdf [file jp2c00994_si_001.pdf]

Supporting Information to "The role of adatoms for the adsorption of F4TCNQ on Au(111)"

Richard K. Berger[1], Andreas Jeindl[1], Lukas Hörmann[1], Oliver T. Hofmann[1*]

[1]Institute of Solid State Physics, Graz University of Technology, 8010 Graz, Austria

* Corresponding Author: o.hofmann@tugraz.at

1 Numerical convergence tests

1.1 Cutoff radius convergence

For numerical reasons, a cutoff potential ensures that each basis function goes to zero after a defined cutoff radius. The radial onset of 6 Å for the cutoff potential was chosen. The onset radius of the cutoff potential was obtained by converging it with respect to the adsorption energy of F4TCNQ on an Au (111) surface that provides the adatom in the experimentally determined $\begin{pmatrix} 5 & 2 \\ 1 & 3 \end{pmatrix}$ surface supercell (see Figure 1a). The results of this convergence test are illustrated in Figure S1.

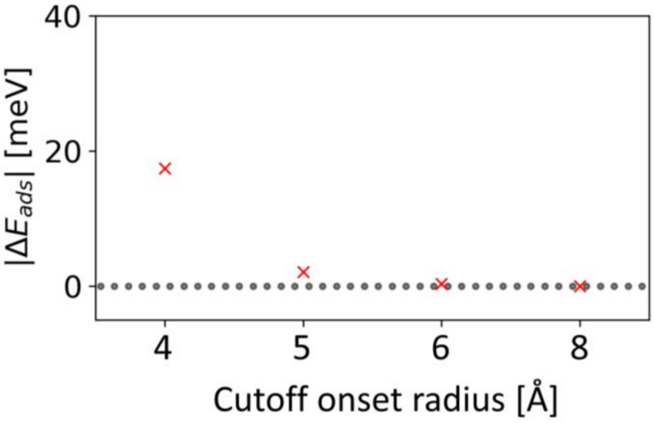

Figure S1: Convergence test for the potential cutoff onset radius performed for F4TCNQ adsorbed within the $\begin{pmatrix} 5 & 2 \\ 1 & 3 \end{pmatrix}$ Au (111) surface supercell with adatoms (see Figure 1a). The y-axis specifies the change in the F4TCNQ adsorption energy according to Eq.: 1 with respect to the value obtained for a cutoff radius of 8 Å.

1.2 K-grid convergence

For periodic calculations, Γ-centered k-grids that follow the directions of the reciprocal lattice vectors of the according systems were used. The number of k-points along the reciprocal axes of the first Brillouin-zone (BZ) was also converged with respect to the adsorption energy (Eq.: 1) within the $\begin{pmatrix} 5 & 2 \\ 1 & 3 \end{pmatrix}$ surface supercell (see Figure 1a), analogue to the cutoff potential described above. The results of the k-grid convergence test can be found in Figure S2.

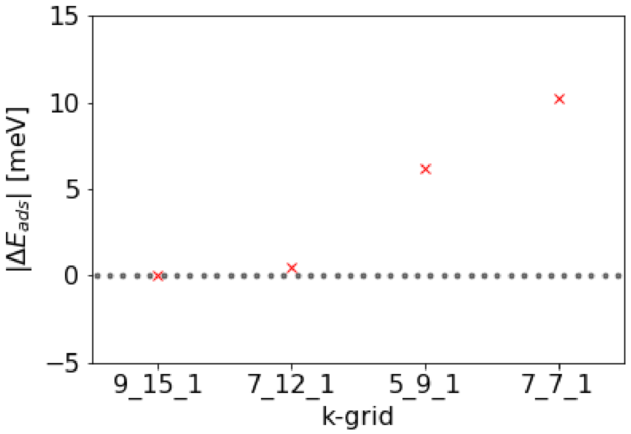

Figure S2: K-grid convergence test for F4TCNQ adsorbed within the $\begin{pmatrix} 5 & 2 \\ 1 & 3 \end{pmatrix}$ Au (111) surface supercell with adatoms (see Figure 1a). The x-axis specifies the number of k-points in the first BZ along the direction of the reciprocal lattice vectors. The y-axis specifies the change in the F4TCNQ adsorption energy according to Eq.: 1 with respect to the value obtained for the 9_15_1 k-grid.

In a converged state we obtained a number of 7, 12, and 1 k-points along the according directions in the first BZ of the $\begin{pmatrix} 5 & 2 \\ 1 & 3 \end{pmatrix}$ surface supercell. This corresponds to a k-point density in the directions of the reciprocal lattice vectors of roughly 14 1/$Å^{-1}$. The same k-grid density was assumed to also be sufficient for the $\begin{pmatrix} 8 & 3 \\ 3 & 6 \end{pmatrix}$ supercell which we use for comparison. Consequently, a number of 5, 6, and 1 k-points along the according directions in the first BZ of the $\begin{pmatrix} 8 & 3 \\ 3 & 6 \end{pmatrix}$ unit cell was found.

2 Initial structures for geometry optimization

2.1 Initial geometries without adatom

For the system without adatom in the $\begin{pmatrix} 5 & 2 \\ 1 & 3 \end{pmatrix}$ supercell, four different initial positions were chosen for the geometry optimizations. Initially, the center of the molecule was placed in an atop, bridge, hollow fcc and hollow hcp position. All initial geometries are depicted in Figure S3.

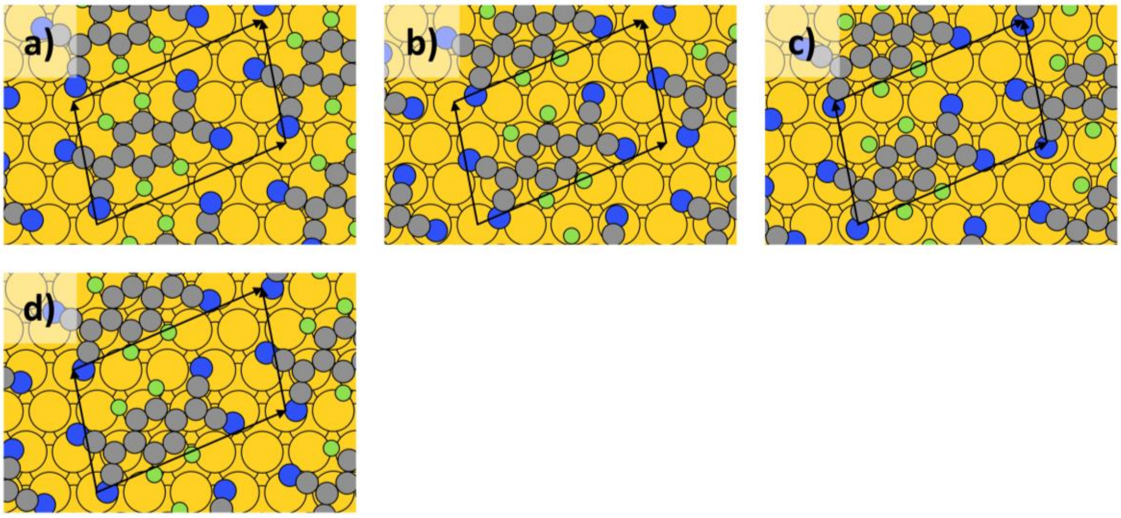

Figure S3: Initial geometries for the geometry optimizations without adatom in the $\begin{pmatrix} 5 & 2 \\ 1 & 3 \end{pmatrix}$ supercell with the central molecular ring ring placed at the atop (a), hollow fcc (b), hollow hcp (c), and bridge (d) positions.

2.2 Initial geometries with adatom

The geometry optimizations for the system with adatom in the experimentally determined $\begin{pmatrix} 5 & 2 \\ 1 & 3 \end{pmatrix}$ supercell were started from six different adsorption positions. The first four (Figure S3a-d) were generated by placing the center of the molecule in an atop, hollow fcc, hollow hcp, or bridge position. The adatom was then placed in the geometric center of the 4

adjacent molecules. Two more geometries (e,f) were generated by shifting the whole molecule-adatom geometry such that the adatom lies either in the fcc hollow or hcp hollow position.

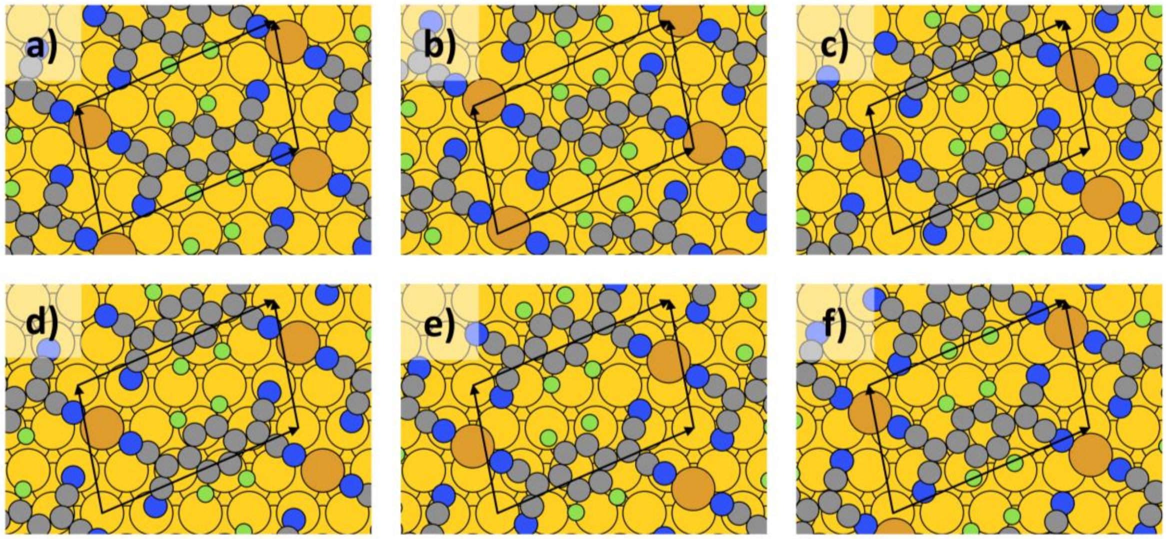

Figure S4: Initial geometries for the geometry optimizations with adatoms in the $\begin{pmatrix} 5 & 2 \\ 1 & 3 \end{pmatrix}$ supercell with the molecule backbone ring placed at the atop (a), hollow fcc (b), hollow hcp (c), and bridge (d) positions, and the adatom placed at the hollow fcc (e) and hollow hcp (f) positions.

3 Stable F4TCNQ adsorption geometries with adatoms

The optimization of the adsorption geometry of F4TCNQ with adatoms in the experimentally determined $\begin{pmatrix} 5 & 2 \\ 1 & 3 \end{pmatrix}$ supercell[1] yields two stable geometries. The adsorption geometry with the adatom located at the bridge surface site of Au(111) (Figure S5b) is energetically less favorable than the geometry with the adatom located at the atop surface site (Figure S5a). The two structures differ in energy by 162 meV.

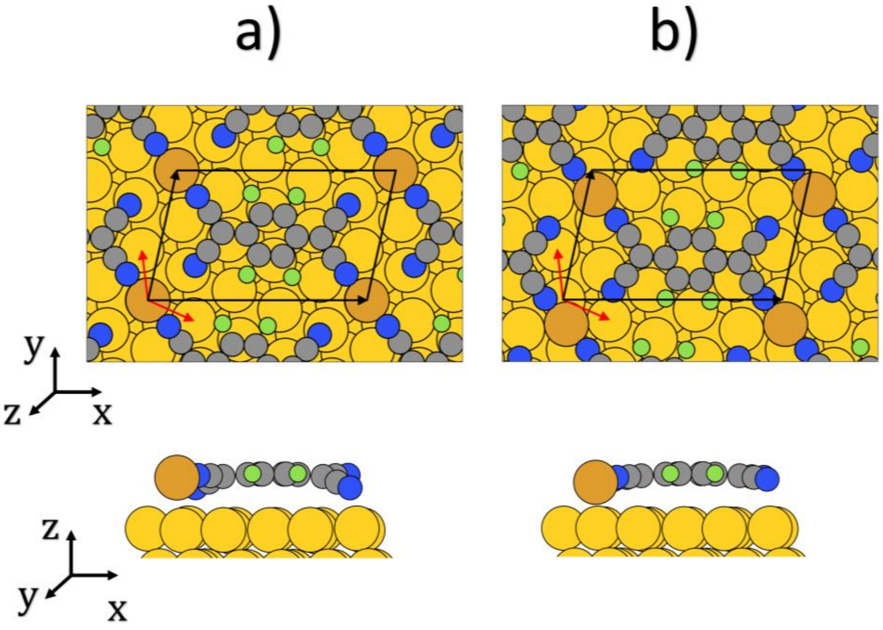

Figure S5: Stable adsorption geometries of F4TCNQ with adatoms within the experimentally determined $\begin{pmatrix}5 & 2\\1 & 3\end{pmatrix}$ supercell[1] (black arrows). Adatoms located at the Au(111) atop surface site (a) and at the Au(111) bridge surface site (b). The primitive unit cell vectors of the Au (111) surface are illustrated by red arrows. The Au adatom is colored orange for simple discrimination to the yellow Au bulk atoms. Carbon, nitrogen and fluorine atoms are colored grey, blue and green, respectively.

The adsorption heights of the atoms in the adlayer depend significantly on the lateral position of the F4TCNQ with respect to the Au(111) surface (see Figure S6).

For the energetically more favorable geometry where the adatom is located at the atop surface site, the quinoid backbone of F4TCNQ is adsorbed 3.23 Å above the substrate surface (Figure S6a). In this case, the adatom, with an adsorption height of 3.03 Å, is lifted up from the substrate surface almost to the same level as the F4TCNQ quinoid backbone.

For the energetically less favorable adsorption geometry, where the adatom is at the bridge site of the Au(111) surface, the adatom adsorption height (2.73 Å) is significantly lowered compared to the energetically ideal structure. In this case, the twist of the F4TCNQ molecule that can be observed both for the adsorption geometry without adatoms (see Figure 1b and Figure S8) and for the energetically ideal adlayer structure with adatoms, is significantly reduced.

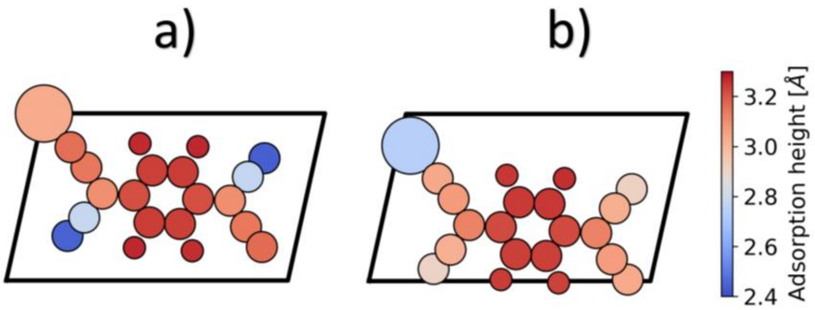

Figure S6: Adsorption heights for the stable adlayer adsorption geometries within the experimentally determined $\begin{pmatrix} 5 & 2 \\ 1 & 3 \end{pmatrix}$ supercell[1] (black cell): adatom located at the Au(111) atop surface site (a), adatom located at the Au(111) bridge surface site (b).

4 Geometry optimization of the adatom on the Au(111) surface

In order to verify the ideal adsorption geometry of native Au adatoms on the Au(111) surface, a geometry optimization for only the adatoms was performed without any additional organic adsorbates. The same $\begin{pmatrix} 5 & 2 \\ 1 & 3 \end{pmatrix}$ surface supercell as for the F4TCNQ adsorption was used. In this supercell the distance between neighboring adatoms is far enough to assume interaction free adatoms.

We find that when only F4TCNQ is present at the Au(111) surface, the hollow surface site is the energetically most favorable position for the adatom to adsorb. This is intuitive since in this position the adatom has the highest possible coordination number to the Au(111) surface atoms.

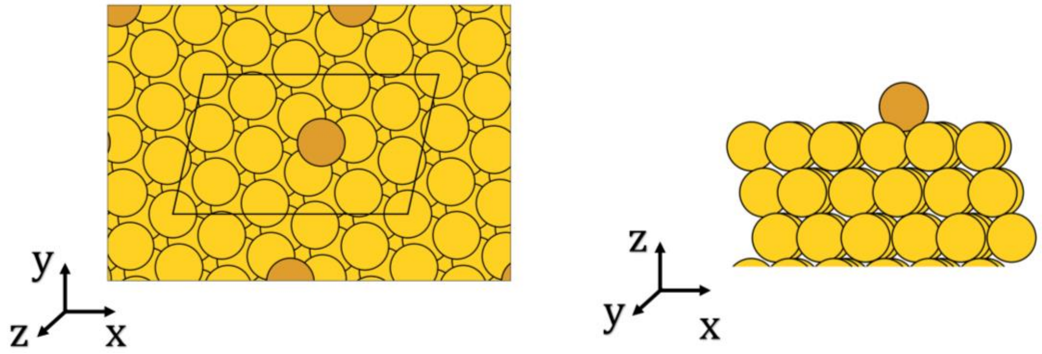

Figure S7: Ideal adsorption geometry of a native Au adatom on the Au(111) surface hollow site in top and side view.

5 F4TCNQ adsorption geometries without adatoms

To assert that the registry is not a consequence of intermolecular interactions, the structure without adatoms was also optimized within a large $\begin{pmatrix} 8 & 3 \\ 3 & 6 \end{pmatrix}$ supercell to simulate very low coverage.

Figure S8a and b show the local adsorption geometries of F4TCNQ without adatoms in the large artificial $\begin{pmatrix} 8 & 3 \\ 3 & 6 \end{pmatrix}$ supercell and within the experimentally determined $\begin{pmatrix} 5 & 2 \\ 1 & 3 \end{pmatrix}$ supercell[1], respectively.

The adsorption geometries of F4TCNQ without adatoms are similar in both unit cells used for the geometry optimization. To quantify this similarity, the distance between equivalent atoms in the two different supercells was calculated for all atoms of the adsorbed F4TCNQ molecule (
Figure S8c). It shows that the maximum displacement of a single atom due to the different adsorption geometries is in the range of 0.1 Å. Therefore, in the main manuscript, we only refer to the structures obtained from the $\begin{pmatrix} 5 & 2 \\ 1 & 3 \end{pmatrix}$ supercell when comparing the cases with and without adatoms.

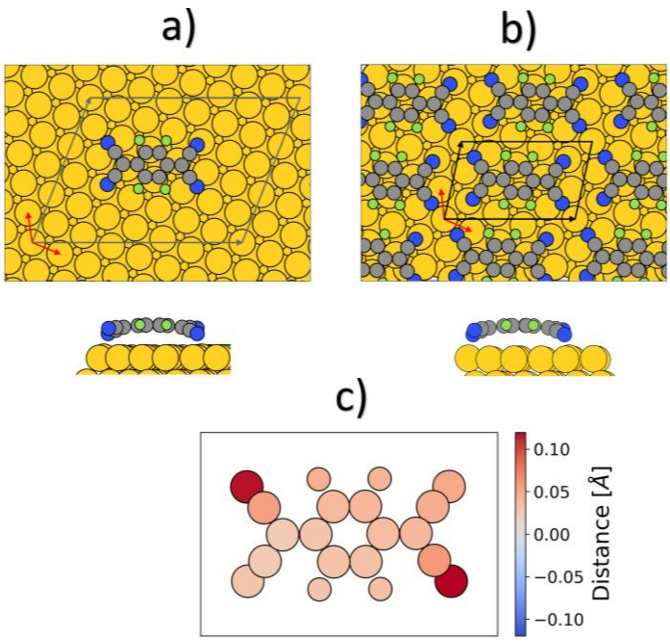

Figure S8: Adsorption geometries of F4TCNQ without adatoms in a large artificial $\begin{pmatrix} 8 & 3 \\ 3 & 6 \end{pmatrix}$ supercell depicted by grey arrows (a) and in the experimentally determined $\begin{pmatrix} 5 & 2 \\ 1 & 3 \end{pmatrix}$ supercell[1] represented by black arrows (b). The primitive unit cell vectors of the Au(111) surface are illustrated by red arrows. The Au bulk atoms are colored yellow. Carbon, nitrogen and fluorine atoms are colored grey, blue and green, respectively. (c) shows the distance between atom positions for the $\begin{pmatrix} 5 & 2 \\ 1 & 3 \end{pmatrix}$ and $\begin{pmatrix} 8 & 3 \\ 3 & 6 \end{pmatrix}$ adsorption geometries.

6 Calculating the bond dipole

As an alternative to the molecular occupation and MODOS, we also calculated the charge transfer from the electron density change caused by the adsorption process. This method is already established,[2,3] for the sake of completeness we still explain the mathematical procedure.

As for the other methods, we compare the cases with and without adatoms. Therefore, the electron density was derived for the complete systems including the adlayer and the Au substrate, depicted in Figure 1. Also, the electron density was calculated for the substrate and the adlayer alone. The adatom was ascribed as part of the substrate in the case where the corresponding complete system features adatoms in the adlayer.

The electron densities of the slab and the adlayer ($\rho_{Slab(\mathbf{r})}$ and $\rho_{F4TCNQ(\mathbf{r})}$, respectively) were subtracted from the electron density of the complete system ($\rho_{System(\mathbf{r})}$). According to

Eq.: S1, the resulting electron density change ($\Delta\rho_{(\mathbf{r})}$) was averaged within the area of a unit cell in the lateral direction of the interface (A_{unit}) yielding the macroscopic electron density change ($\Delta\rho_{(z)}$), depending only on the coordinate of the surface normal.

$$\Delta\rho_{(z)} = \frac{1}{A_{unit}} \iint_{A_{unit}} \Delta\rho_{(\vec{r})}\, dx\, dy \qquad \text{Eq.: S1}$$

$$\Delta\rho_{(\vec{r})} = \rho_{System\,(\vec{r})} - \rho_{Slab\,(\vec{r})} - \rho_{F4TCNQ(\vec{r})}$$

The resulting macroscopic electron density change ($\Delta\rho_{(z)}$) caused by the adsorption of F4TCNQ, is illustrated in

Figure S9a, for both the case with and without adatoms. To obtain the interface charge transfer (ΔQ), i.e., the charge per unit cell area that is transferred along the surface normal, the macroscopic electron density change ($\Delta\rho_{(z)}$) was integrated along the surface normal coordinate and multiplied by the lateral unit cell area A_{unit}, according to Eq.:S2.

$$\Delta Q_{(z)} = A_{unit} \int_{-\infty}^{z} dz'\, \Delta\rho_{(z')} \qquad \text{Eq.:S2}$$

Figure S9b compares the interface charge transfer ΔQ for the two systems with and without adatoms.

In case there is no adatom present, one finds charge transfer from the metal to the adlayer (

Figure S9b$_1$). This is characterized by the rapid drop of ΔQ in the regime of the metal surface and the gradual increase of ΔQ from the metal surface towards the adlayer. On the contrary, when adatoms are involved, there is no monotonic increase of ΔQ from the metal surface towards the interface, i.e., there's no significant net charge transfer from the metal towards the

adlayer. Consequently, the charge transfer induced surface dipole is significantly reduced in case adatoms are part of the adlayer. We can only observe a local shift in the charge density but no gradual charge transfer from the substrate to the adlayer. Due to this locality of the charge shift, there is little effect on the overall surface dipole, compared to the case without adatoms.

Figure S9c shows the resulting bond dipole (BD) potential jumps, $\Delta\phi_{BD}$, calculated from the electron density variation, according to Eq.: S3.

$$\Delta\phi_{BD(z)} = e/\epsilon_0 \int_{-\infty}^{z} dz' \int_{-\infty}^{z'} dz''\, \Delta\rho_{(z'')} \qquad \text{Eq.: S3}$$

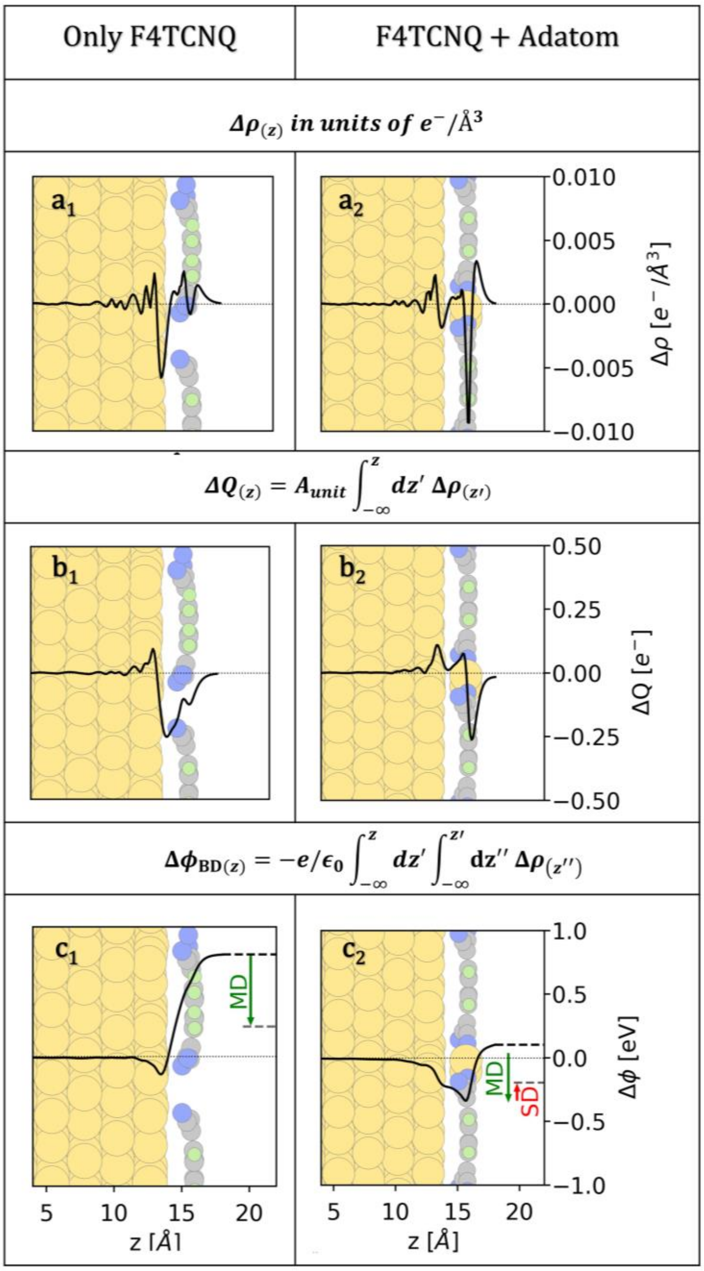

Figure S9: Interface charge density change caused by F4TCNQ adsorption: a) Variation in the local charge density due to F4TCNQ adsorption. b) Interface charge transfer per lateral unit cell area A_{unit} c) Potential jump due to charge transfer at the interface (BD). Permanent molecular dipoles (MD) and substrate dipoles (SD) are indicated by green and red arrows, respectively.

7 Frontier orbitals of the Adatom and Molecule

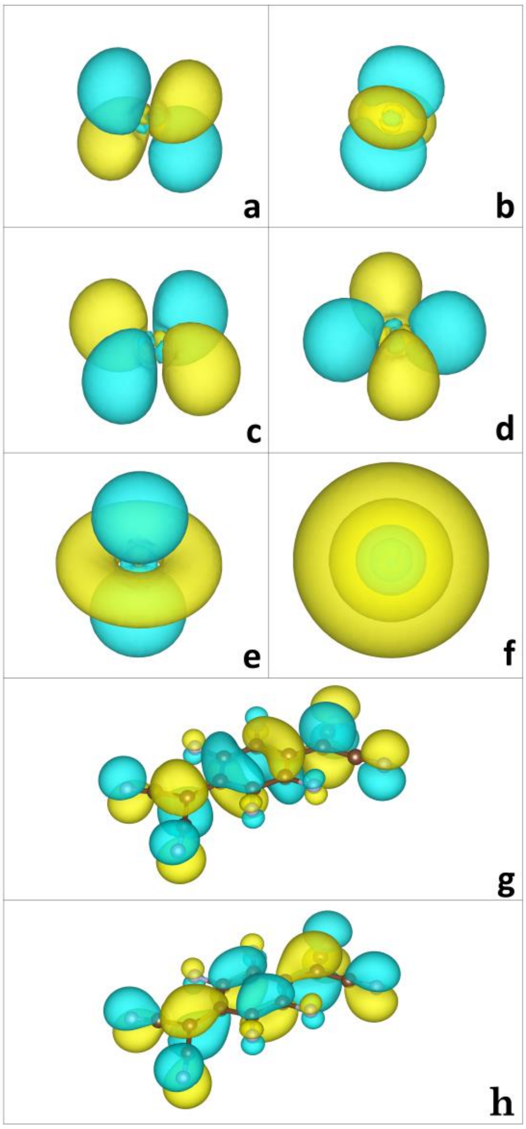

Figure S10: Atomic orbitals of Au: a) 5d_{xz}, b) 5d_{yz}, c) 5d_{xy}, d)5d_{x2y2}, e) d_z, f) 6s, Molecular orbitals of F4TCNQ; g) LUMO, h) HOMO

8 References

1. Faraggi MN, Jiang N, Gonzalez-Lakunza N, et al. Bonding and charge transfer in metal-organic coordination networks on Au(111) with strong acceptor molecules. *J Phys Chem C*. 2012;116(46). doi:10.1021/jp306780n

2. Heimel G, Romaner L, Zojer E, Bredas JL. Toward control of the metal-organic interfacial electronic structure in molecular electronics: A first-principles study on self-assembled monolayers of π-conjugated molecules on noble metals. *Nano Lett*. 2007;7(4). doi:10.1021/nl0629106

3. Heimel G, Romaner L, Brédas JL, Zojer E. Interface energetics and level alignment at covalent metal-molecule junctions: π-conjugated thiols on gold. *Phys Rev Lett*. 2006;96(19). doi:10.1103/PhysRevLett.96.196806
